# *In-situ* ATR-FTIR for dynamic analysis of superhydrophobic breakdown on nanostructured silicon surfaces

**DOI:** 10.1038/s41598-018-30057-w

**Published:** 2018-08-02

**Authors:** Nandi Vrancken, Jiaqi Li, Stefanie Sergeant, Guy Vereecke, Geert Doumen, Frank Holsteyns, Chang Chen, Herman Terryn, Stefan De Gendt, XiuMei Xu

**Affiliations:** 1 Research Group Electrochemical and Surface Engineering (SURF), Dept. of Materials & Chemistry (MACH), Vrije Universiteit Brussel (VUB), Pleinlaan 2, B-1050 Brussels, Belgium; 2 Imec, Kapeldreef 75, 3001 Leuven, Belgium; 3 Department of Physics and Astronomy, KU Leuven, Celestijnenlaan 200D, 3001 Leuven, Belgium; 4 KU Leuven, Celestijnenlaan 200, 3001 Leuven, Belgium

## Abstract

Superhydrophobic surfaces are highly promising for self-cleaning, anti-fouling and anti-corrosion applications. However, accurate assessment of the lifetime and sustainability of super-hydrophobic materials is hindered by the lack of large area characterization of superhydrophobic breakdown. In this work, attenuated total reflectance−Fourier transform infrared spectroscopy (ATR-FTIR) is explored for a dynamic study of wetting transitions on immersed superhydrophobic arrays of silicon nanopillars. Spontaneous breakdown of the superhydrophobic state is triggered by *in-situ* modulation of the liquid surface tension. The high surface sensitivity of ATR-FTIR allows for accurate detection of local liquid infiltration. Experimentally determined wetting transition criteria show significant deviations from predictions by classical wetting models. Breakdown kinetics is found to slow down dramatically when the liquid surface tension approaches the transition criterion, which clearly underlines the importance of more accurate wetting analysis on large-area surfaces. Precise actuation of the superhydrophobic breakdown process is demonstrated for the first time through careful modulation of the liquid surface tension around the transition criterion. The developed ATR-FTIR method can be a promising technique to study wetting transitions and associated dynamics on various types of superhydrophobic surfaces.

## Introduction

Inspired by nature, numerous biomimetic functional materials with controlled wettability have been designed. Examples include superhydrophobic anti-fouling surfaces for medical purposes^1^, drag reduction^2^ and corrosion protection^3,4^, transparent self-cleaning surfaces for photovoltaics^5^ and microfluidic devices with selective wetting property^6^. Sustainability of superhydrophobic surfaces has become a critical issue that limits the application and lifetime of these functional materials and devices. One of the common causes for superhydrophobic breakdown comes from pollution. The presence of detergents and antibiotics in rivers as well as oil spills in marine environment can severely impact the surface tension of water^7–9^. Reduction of the surface tension by more than 25% has been reported for some wastewater streams^10^ and in the presence of some bacteria species that excrete tensio-active substances, e.g. pseudomonas strains^11^. As a result, the superhydrophobic anti-fouling and anti-corrosion coatings on ship hulls can lose their effectiveness^12^, and the diving performances of several aquatic bird species are deteriorated due to the loss of water repellence of their plumage^13^. Clearly, a better understanding of the stability and sustainability of superhydrophobic surfaces will have an enormous economic and ecological impact.

The breakdown of superhydrophobic states can take place spontaneously upon gravity^14^, vibration^15,16^, evaporation^17,18^, contamination by surfactants^19^ or can be forced by electrowetting^20^ or external pressure^15,21,22^. Extensive studies have been carried out to investigate the stability of superhydrophobic states and energy barriers for wetting transitions on different types of surfaces^13,14,23–32^. However, a very wide range of critical pressures^15,21,22,30,33–36^ and wetting velocities^23–25,34^ has been reported in the literature. Using transparent substrates patterned with micropillars, the dynamical process of superhydrophobic breakdown from a local infiltration point was analyzed by high speed imaging^23–25^. It has been found that wetting kinetics are greatly slowed down when the intrinsic contact angle θ is close to the critical contact angle θ_c_, due to the vanishing energy gain for wetting. Therefore, experimental assessment of energy barriers and breakdown dynamics should be correlated to the deviation from this transition criterion, which is still lacking in most work. Besides, for real-life applications, several infiltration spots, possibly triggered by local defects, may appear simultaneously on immersed surfaces and result in multiple moving wetting fronts. Thus, it is of utmost importance to characterize the critical wetting transition criterion and wetting dynamics for large surface areas.

Classical models predict that structured surfaces made from inherently hydrophilic materials (intrinsic contact angle on flat surfaces *θ* < 90°) will be fully wetted, whereas surface patterning may turn hydrophobic materials (*θ* > 90°) superhydrophobic. The transition criterion from superhydrophobic wetting (the Cassie-Baxter state) to complete wetting (the Wenzel state) solely depends on the geometrical characteristics of the patterned surface:

1 $$\,{\theta }_{c}^{CB-W}=co{s}^{-1}(\frac{{f}_{s}-1}{r-{f}_{s}})$$

where, the surface roughness r is defined as the ratio of the actual total surface area and the projected surface area, and f_s_, the solid surface fraction as the ratio of the top surface area and the projected area^37^. ${\theta }_{c}^{CB-W}$ is the critical contact angle as measured on a flat surface of the same material below which a transition to the Wenzel state will occur. Significant deviations from classical models can be found in literature. Cassie-Baxter states have been reported even for intrinsically hydrophilic materials with contact angles in the range of 70–90°^37–41^. These discrepancies hamper accurate prediction of the performance and lifetime of superhydrophobic surfaces in real applications.

Only few techniques have been demonstrated for accurate determination of wetting transitions and *in-situ* kinetic study of the breakdown process. Contact angle measurements are the most commonly used methods in experimental studies, but they cannot provide detailed information on the progress of superhydrophobic breakdown. A few optical or acoustic based techniques have been developed to characterize wetting states more accurately at the microscale^17,34,42–46^. For example, the solid-liquid interface and contact line motions upon wetting/dewetting can be visualized using confocal microscopy^42,44,45^. In addition, interference microscopy is applied to reconstruct the 3D liquid interface and analyze local liquid penetration^34^. Use of nanostructured surfaces instead of micro-architectures improves the underwater stability and antifouling performance^26,47^. Energy calculations reveal that critical pressures for superhydrophobic breakdown on nanostructures can be 2–3 orders of magnitude higher than for microstructures^26,29^. However, wetting characterization at the nanoscale is even more challenging and advanced techniques, such as environmental^48^- or cryo^49^- SEM, are required to visualize the liquid-solid interface. Recently we have successfully applied different techniques to characterize wetting properties of nanopatterned silicon substrates^37,41,50–52^. Evaluation of the acoustic reflection coefficient yields quantitative information on transition criteria^51^. More accurate quantification of nanoscale liquid imbibition is provided by optical reflectance spectroscopy^37,50^, although complex modelling is required to interpret the spectra. Both techniques show some potential for *in-situ* kinetic studies, but they are limited to local area characterization (spot size ~50–500 µm). For real applications, it is desirable to monitor the average wetting state over a macroscopic area to assess the overall breakdown kinetics.

In this paper, we use ATR-FTIR spectroscopy for a systematic *in-situ* investigation of wetting states and wetting transitions on superhydrophobic silicon nanostructures with varying aspect ratio. ATR-FTIR is used extensively in many applications that require surface-sensitive and/or non-destructive characterization^53–56^. When an infrared (IR) beam is totally reflected on the inner surface of an ATR crystal, an evanescent wave is generated and penetrates the sample in contact with the crystal. The sample molecules absorb the IR radiation with wavelengths corresponding to their characteristic vibration modes. In a previous work^41^, we demonstrated the potential of monitoring the relative intensity of water absorption bands for large-area wetting characterization. In this work, the technique is extended to study the dynamic process of superhydrophobic breakdown. First, an estimation of the critical contact angle and surface tension for superhydrophobic breakdown is obtained with contact angle measurements and more accurately determined with ATR-FTIR. Spontaneous wetting of nanostructures starts once the surface tension of the liquid is reduced below a critical value, and the breakdown process is measured in real time by ATR-FTIR. Then we demonstrate that an excellent control of superhydrophobic breakdown can be achieved by *in situ* variation of liquid’s surface tension. Wetting kinetics and breakdown mechanism will be discussed at the end.

## Methods

### Test structures

Silicon wafers are patterned with square-packed silicon nanopillar arrays using deep UV immersion lithography and plasma etching as detailed in previous works^37,41,57–59^. Pillar heights range from 75 nm to 265 nm with aspect ratios starting from approximately 2.5 up to 8. Detailed geometrical characteristics of the different structures are determined from Matlab fitting of XSEM-images and are tabulated in Table 1. The samples are subjected to dry surface modification steps to render the surface superhydrophobic. After UV/O_3_-treatment for 15 minutes to remove any organic contamination, the pillars are grafted with 1H,1H,2H,2H-perfluorodecyl-trichlorosilane (FDTS, supplied by ABCR GmbH with a purity of 97 mol%) which increases the static water contact angle to approx. 149° on patterned surfaces and approx. 112 ± 2° on flat surfaces.

**Table 1:** Test structure dimensions.

Structure	Height (nm)	Pitch (nm)	Diameter (nm)	r	f_s_	θ_c_ (°)
1	78 ± 9	90	29 ± 3	1.9 ± 0.2	0.082 ± 0.02	121
2	150 ± 9	90	35 ± 3	3.0 ± 0.3	0.12 ± 0.02	108
3	260 ± 9	90	40 ± 3 (center) 45 ± 3 (top)	5.4 ± 0.4	0.20 ± 0.03	99
4	265 ± 9	90	33 ± 3	4.4 ± 0.4	0.11 ± 0.02	102

θ_c_ is the critical contact angle for the transition from Cassie-Baxter to Wenzel state and is calculated based on the geometrical characteristics of the structures, as given by equation (1). The solid surface fraction, f_s_, is defined as A_solid_/A_projected_ where A_solid_ is the total area covered by the pillar tips and A_projected_ is the projected surface area. The surface roughness, r, is the ratio of the actual surface area and the projected surface area: r = A_real_/A_projected_.

### Contact angle measurements

Contact angle measurements are performed on an OCA200 measurement instrument under controlled temperature (22 ± 1 °C) and relative humidity (40 ± 10%) conditions. Droplet size is 1 µl for hydrophilic and slightly hydrophobic samples. In the case of superhydrophobic samples, the droplet size was increased to 2.5 µl to allow the drop to detach from the needle.

### ATR-FTIR

**Figure 1:**
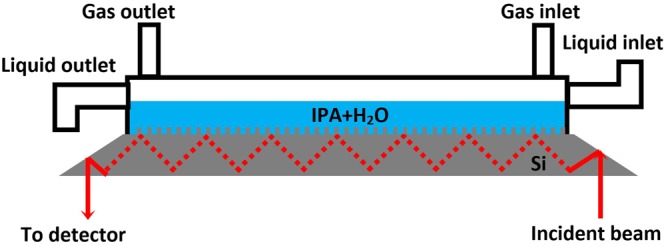
Schematic illustration of the liquid cell for ATR-FTIR. The image is not to scale. Dynamic wetting tests are performed using a superhydrophobic crystal that is entirely covered with liquid. Bubblers can be connected to the cell via the ‘gas inlet’ connection.

ATR-crystals (crystal dimensions: 5.5 × 3.0 cm^2^, thickness 775 μm, angle of incidence 60°) are prepared from the patterned wafers by mechanical polishing. The crystals are then mounted in a customized flow cell and positioned on a Nicolet 6700 FT-IR spectrometer. The cell is schematically illustrated in Fig. 1. IR-light, entering from one side of the crystal, is internally totally reflected 18 times before leaving the crystal and being collected in the detector. An evanescent wave is generated at every point of reflection, which penetrates the sample and is absorbed by the sample molecules. As the pillars are directly patterned into the surface, sample contact issues are eliminated and sensitivity is enhanced. At first, a background of the dry crystal in air is recorded with a resolution of 1.0 cm^−1^ and 100 scans. This background is subtracted from all recorded experimental spectra to cancel out backside reflection and eliminate contributions from the tool and the atmosphere. Next, liquid is injected into the liquid cell and a spectrum is recorded with 4–6 scans at the same resolution as the background spectrum. Sampling time is approximately 6–9 seconds.

The environmental cell is connected to two bubblers, one filled with deionized water and one filled with pure isopropyl alcohol (IPA, Cleanroom^®^ LP grade purchased from KMG chemicals). By switching between the two bubblers using a three-way valve, a controlled flow of nitrogen saturated with IPA or water can be sent through the cell. This set-up allows for *in-situ* variation of the liquid composition through condensation of IPA vapor or water vapor at the interface between ambient and liquid. For wetting dynamics experiments the IPA concentration was increased *in situ* by flowing IPA-saturated N_2_ gas through the cell until a targeted liquid concentration was reached. Next, the tubing was flushed with dry N_2_ for 5 min after which all flows were switched off and the cell was left to stabilize. This procedure ensures the IPA-saturated cell atmosphere is replaced by dry N_2_ and no more IPA condensation takes place on the surface of the liquid. As the cell is closed, an equilibrium will be established between gas and liquid and the liquid composition remains constant. A similar approach is used for the wetting actuation experiments, but involves a flow of water saturated N_2_ gas in the cell instead of stabilization. Standard flow rates are 35 ccm for all experiments, unless mentioned otherwise. The instantaneous IPA-concentration around the pillars can be directly extracted from the spectrum based on the relative intensity ratio of the CH-stretching band at 2930 cm^−1^ and the OH-stretching band at 3380 cm^−1^. A calibration curve was established in our previous work^41^ using premixed solutions with known liquid composition and a linear correlation was found between I_CH-stretch_/I_OH-stretch_ and the IPA concentration with a measurement error of ± 0.1 mol% in the range of interest (2–4 mol%). A minor correction is applied to account for the relative attenuation between both peaks in Cassie-Baxter state and mixed wetting states. Different concentration profiles can be achieved without notably affecting the relative intensity ratio of the water bands (example of a concentration profile and corresponding intensity ratio in Supplementary Fig. S1).

### FDTD simulations

Full 3D finite-difference time-domain (FDTD) analysis of evanescent wave attenuation was performed using the commercial Lumerical FDTD Solutions solver. A cylindrical silicon nanopillar on a flat silicon substrate is either fully embedded in water (Wenzel state) or in a composite state of air and water (Cassie-Baxter state). The diameter and height of pillars were set by using the experimental SEM measurement data. The refractive index of crystalline silicon was taken from Handbook of Optical Constants of Solids by Edward Palik^60^. Bloch and perfectly matched layer boundary conditions were employed in the directions that are parallel and perpendicular to the axis of the nanopillar, respectively. A plane wave with an incidence angle of 60° was used to excite the silicon nanopillar. A frequency-domain field profile monitor and a refractive index monitor were employed in order to correctly extract the electric field intensity in water. A uniform 2 nm override mesh was also used over the whole simulation volume. Electric field distribution within the range of 2 µm away from the nanopillared silicon substrate was simulated.

## Results and Discussion

### Contact angle measurements

**Figure 2:**
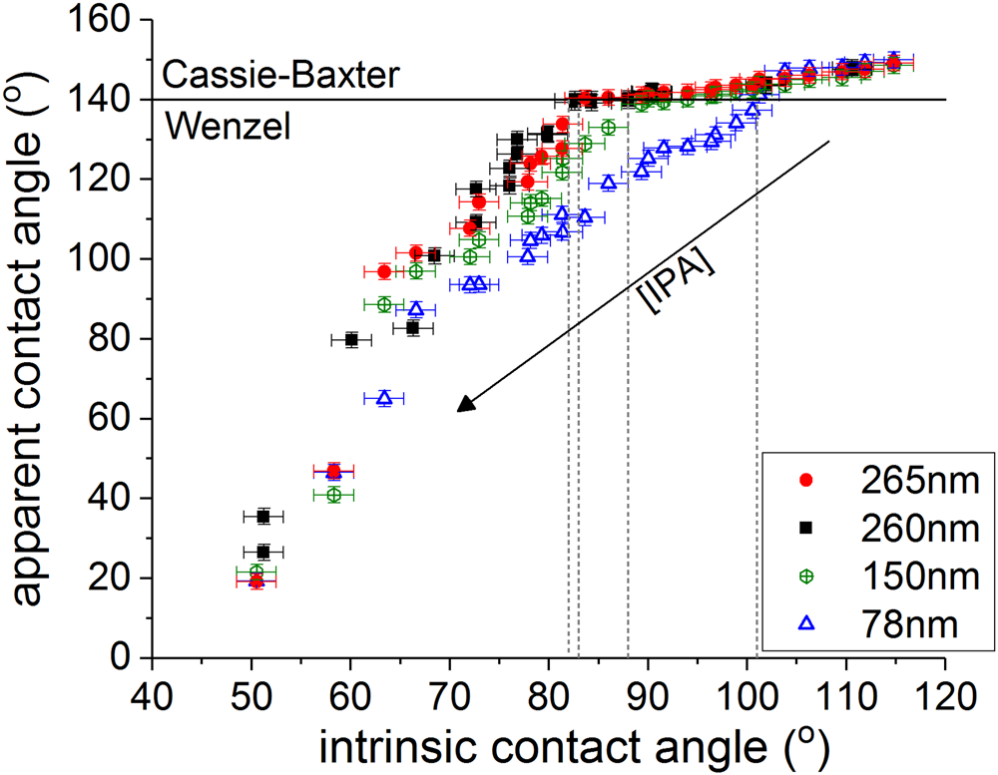
Apparent contact angles measured on the FDTS-coated patterned surface as a function of the intrinsic contact angles measured for the same liquid on a FDTS-coated flat silicon surface. The horizontal black line marks the approximate transition from Cassie-Baxter to Wenzel wetting. The vertical grey lines represent the corresponding critical contact angles for the different structures.

Goniometric measurements are used to obtain a first estimation of the wetting transition criterion for structures with different dimensions. Droplets of deionized water exhibit a contact angle of 149 ± 2° on all structures, corresponding to the Cassie-Baxter state. The superhydrophobic character has already been confirmed in previous works for similar nanostructures using various analysis methods^37,41,51^. The liquid surface tension is gradually lowered by injecting premixed mixtures of water and isopropanol (IPA) with increasing IPA concentrations. The apparent contact angles, as measured on the patterned surfaces, are plotted in Fig. 2 as a function of the intrinsic contact angles on flat samples with the same surface termination. For apparent contact angles above 140°, all four structures exhibit similar behavior and the contact angle shows only minor dependence on the IPA concentration. This region corresponds to the Cassie-Baxter state. Upon gradually reducing the liquid surface tension by further increasing the IPA concentration, a sharp discontinuity is observed and the contact angle drops steeply with a much larger slope. This region corresponds to the Wenzel regime and the transitions are indicated with grey dashed lines. Breakdown of the Cassie-Baxter state occurs when the intrinsic contact angle *θ* is reduced below 101° ± 2°, 88 ± 2°, 82° ± 2° and 83° ± 2° for pillar heights of 78, 150, 260 and 265 nm, respectively. Note that these critical contact angles are much lower than the theoretical predictions given in Table 1.

Contact angle measurements only yield information on the wetting behavior near the edge of the droplet and are therefore less accurate for analysis of wetting transitions or partial wetting^61^. We applied ATR-FTIR to obtain a more accurate determination of the transition criterion and elaborate on the dynamic aspects of the wetting transition.

### ATR-FTIR

**Figure 3:**
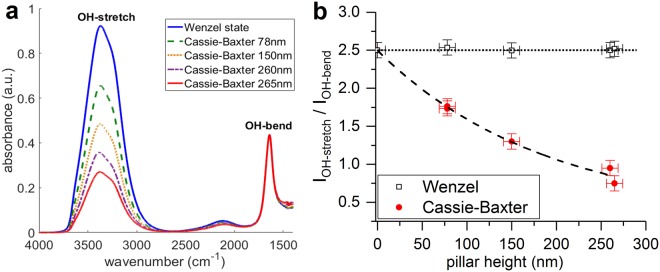
(**a**) FTIR spectra of pure water in Wenzel state on a hydrophilic patterned crystal (blue) and in Cassie-Baxter state on superhydrophobic FDTS-coated pillars with heights of 78 nm (green), 150 nm (orange), 260 nm (purple) and 265 nm (red). The spectra are corrected for the background and are normalized based on the OH-bending peak. (**b**) Relative peak intensity ratio of the water stretching and bending bands as a function of pillar height for hydrophilic (UV/O_3_-treated, Wenzel state) and superhydrophobic (FDTS-coated, Cassie-Baxter state) crystals.

ATR-crystals are prepared for all test structures and mounted in the liquid cell on the FT-IR spectrometer. If liquid fully wets the silicon nanostructures (Wenzel wetting), the obtained spectrum is similar to the ones recorded on an unpatterned crystal. An example spectrum of pure water in Wenzel state is plotted in Fig. 3a, and compared to the normalized spectra of pure water on various superhydrophobic structures. Water exhibits two characteristic bands^62^, i.e. the OH-stretching band (3380 cm^−1^) and the OH-bending band (1640 cm^−1^) with a relative peak intensity ratio of 2.5 ± 0.1, as calculated after baseline correction for both water peaks and normalization with respect to the OH-bending band. The obtained relative peak intensity ratios of the water bands are plotted in Fig. 3b for different hydrophilic (UV/O_3_-treated) and superhydrophobic (FDTS-coated) structures. In Wenzel state, this ratio is nearly independent of the structure dimensions, as water is in direct contact with the bottom silicon surface where IR light is totally reflected. However, the intensity ratio is clearly reduced on superhydrophobic pillars and shows a strong dependence on the structure height. The evanescent wave is attenuated by the air layer in between the structures before reaching the liquid on top. As discussed in previous works^41^, the attenuation is wavelength dependent, and the OH-stretching band at higher wavenumbers is more attenuated by this air layer. Consequently, not only the overall spectrum intensity decreases, but also the relative peak intensity ratio of the water bands is altered. Unlike the absolute peak intensity, this relative intensity ratio is independent of the total immersed area and is not affected by experimental variations. Therefore, it is an excellent measure for the average wetting state over large areas.

**Figure 4:**
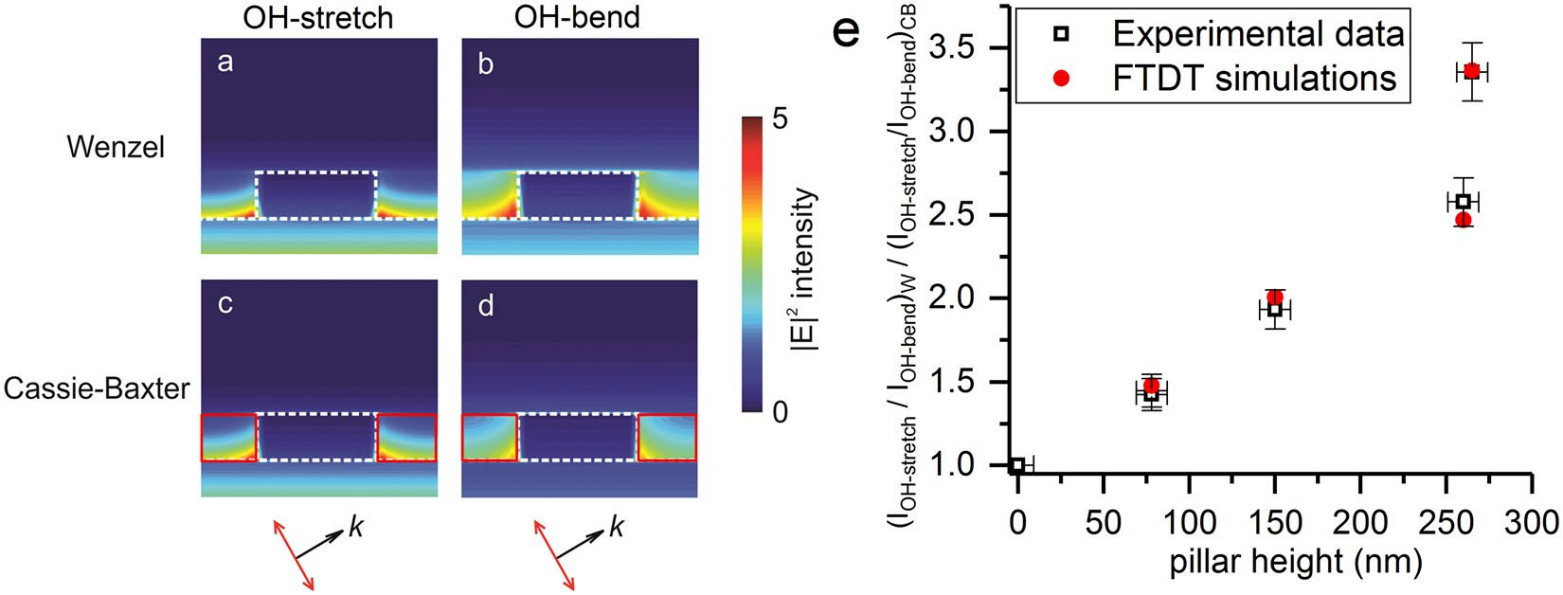
(**a–d**) Electric field intensity distributions in the cross section through the center of the silicon nanopillar for the OH-stretching and OH-bending bands at different wetting states. White dashed lines indicate the silicon boundary. Volume elements occupied by air are indicated in red and are not considered for calculation of the total intensity from water absorption. (**e**) Comparison of the FDTD simulations and experimental data of the change in relative intensity ratio resulting from a wetting transition for different pillars heights. The subscripts W and CB stand for Wenzel and Cassie-Baxter states, respectively. Ratios are calculated for pure water.

As the ATR-FTIR method is based on the wavelength-dependent attenuation of evanescent waves, the sensitivity depends on the thickness of the air layer, which is directly related to the pillar height. If the pillar height increases, the air layer becomes thicker and the differential attenuation is more pronounced, resulting in a low(er) relative peak intensity ratio as shown in Fig. 3b. In order to better understand the application range of the method, attenuations of evanescent waves for different structure heights are investigated theoretically. It is well known that the electric field intensity of an evanescent wave decays exponentially with distance. In 1D approximations, the penetration depth is a measure to quantify the distance an evanescent wave can travel through a material. It is defined as the sample depth for which the intensity of the evanescent wave has decreased by 1/e and can be calculated using following equation^63,64^:

2 $${{\rm{d}}}_{{\rm{p}}}=\frac{1}{2{\rm{\pi }}{\rm{\nu }}\sqrt{{{\rm{n}}}_{1}^{2}{\sin }^{2}{\rm{\beta }}-{{\rm{n}}}_{2}^{2}}}$$

where ν is the wavenumber of the evanescent wave, n_1_ and n_2_ are the refractive indices of the crystal and the sample, respectively and β is the angle of incidence. Penetration depths in air for the OH-bending and OH-stretching bands at 1640 cm^−1^ and 3380 cm^−1^ equal 350 nm and 170 nm, respectively. Actual sampling depths can be approximated by 3d_p_^64^. For structures with heights exceeding the sampling depths, the air layer may already completely attenuate the evanescent waves before it reaches the water on top of the pillars. In that case, a differentiation from the pure Wenzel states can still be made with both water bands vanishing from the spectrum. On the other hand, for very short structures, when the change in absorption intensities becomes comparable to the measurement errors, the two wetting states cannot be distinguished anymore. The simple 1D model of exponential decay of evanescent waves cannot describe the actual attenuation around the silicon nanopillar array. Therefore, 3D FDTD simulations were employed in order to calculate the relative change of the intensity ratio (I_OH-stretching_/I_OH-bending_) in the Cassie-Baxter state with respect to the Wenzel state. Simulation details can be found in the Methods section. 3D electric field intensity distributions around nanopillars have been simulated. In Fig. 4a–d, the electric field intensity profiles in the cross section through the center of the silicon nanopillar are compared at wavenumbers corresponding to the two water absorption bands. For both wetting states, evanescent waves at higher wavenumbers are found to decay faster with distance, indicating more attenuation of the OH-stretching band. In order to quantitatively characterize the water absorption, the total intensity contributing to the absorption bands is calculated as the sum of the electric intensity over all volume elements occupied by water. Since the absorption coefficients are unknown, only the relative change in intensity ratios can be compared with the experimental results. An excellent agreement between simulations and experiments can be found in Fig. 4e. The difference of the intensity ratios between Wenzel and Cassie-Baxter states is less pronounced for shorter structures, but the technique should be sufficiently sensitive for structures with a minimum height of 20–30 nm.

The critical composition of IPA-water mixtures for the onset of superhydrophobic breakdown can be accurately determined by ATR-FTIR. First, a mixture with low IPA concentration was injected in the liquid cell to cover the entire crystal. The initial concentration is well below the critical concentration for wetting transition found with contact angle measurements. Thus, the liquid initially resides in pure Cassie-Baxter state on the crystal. Then the IPA concentration is gradually increased by controlled condensation of IPA vapor until a wetting transition is observed (details in supplementary figure S2). The onset of superhydrophobic breakdown is defined as when the intensity ratio I_OH-stretching_/I_OH-bending_ increases by 0.15 (an arbitrarily defined small value to exclude effects of initial breakdown at borders of the patterned nanopillars). The instantaneous IPA concentration near the nanostructured surface can be accurately determined from the intensity ratio of CH-stretching band (characteristic for IPA) and OH-stretching band (characteristic for water)^41^. The critical intrinsic contact angle is measured on a flat, FDTS-coated crystal using premixed IPA-water mixtures with the same critical composition that leads to spontaneous wetting in the ATR-FTIR experiments.

**Figure 5:**
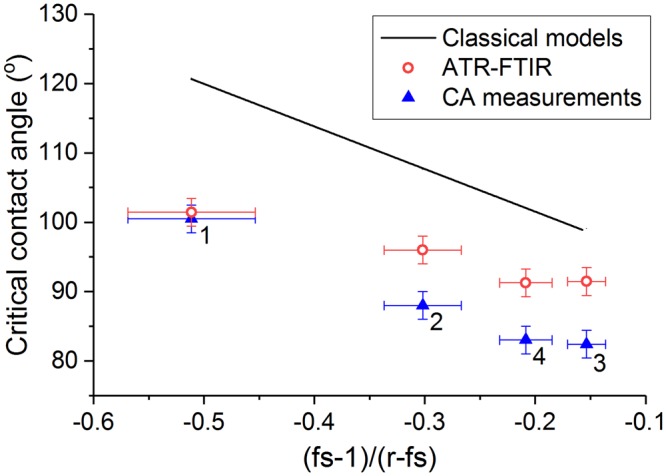
Comparison of the critical contact angles for superhydrophobic breakdown, as determined with contact angle measurements (blue triangles) and ATR-FTIR (red circles) as a function of the geometrical parameters of the test structures. The solid black line corresponds to the predictions of the classical wetting models. The numbers ‘1’, ‘2’, ‘3’ and ‘4’ refer to the structure ID’s, as given in Tables 1 and 2.

Figure 5 and Table 2 summarize the experimentally obtained critical contact angles as a function of the surface roughness and solid surface fraction, compared to the predictions by the classical wetting models (equation (1)). The numbers 1–4 refer to the structure ID’s as tabulated in Table 1. The plotted values for the critical contact angle refer to intrinsic contact angles on a flat silicon surface with FDTS coating. Liquid with a contact angle below the critical angle (thus lower surface tension than the critical surface tension) will show spontaneous breakdown of the superhydrophobic state. A shift towards lower critical contact angles is found for both ATR-FTIR and contact angle measurements compared to the predictions by the classical wetting models. Similar shifts have been observed in previous works^37,52^ and can be attributed to differences in surface state between the pillar side walls and the flat silicon reference. The complex fabrication process of the nanopillars involves multiple (plasma) etching steps that may give rise to surface alterations, including mixed crystal planes and atomic-scale lattice defects. Therefore, the static contact angle measured on a flat reference surface with similar surface termination may not be very representative for the contact angle on the pillar sidewalls, which can cause a shift between classical wetting models and experimental observations. This observation does not compromise the validity of the classical wetting models, although it severely restricts its use due to practical limitations. The differences between contact angle and ATR-FTIR measurements originate from different sensitivities of the characterization techniques. The transition criterion measured from contact angle measurements is closer to the fully wetted Wenzel state, while ATR-FTIR is a surface sensitive technique that can accurately determine the onset of superhydrophobic breakdown. Therefore, ATR-FTIR can be used to study the dynamic breakdown process, which is important for predicting the lifetime of water-repellent and self-cleaning surfaces.

**Table 2:** Critical contact angles for the transition from Cassie-Baxter to Wenzel state, as predicted by the classical models and experimentally determined with contact angle measurements and ATR-FTIR.

Structure	Height (nm)	Θ_c, classical models_ (°)	Θ_c, contact angle_ (°)	Θ_c, ATR-FTIR_ (^o^)
1	78 ± 9	121	101 ± 2	101 ± 2
2	150 ± 9	108	88 ± 2	96 ± 2
3	260 ± 9	99	82 ± 2	91 ± 2
4	265 ± 9	102	83 ± 2	91 ± 2

### Wetting dynamics

**Figure 6:**
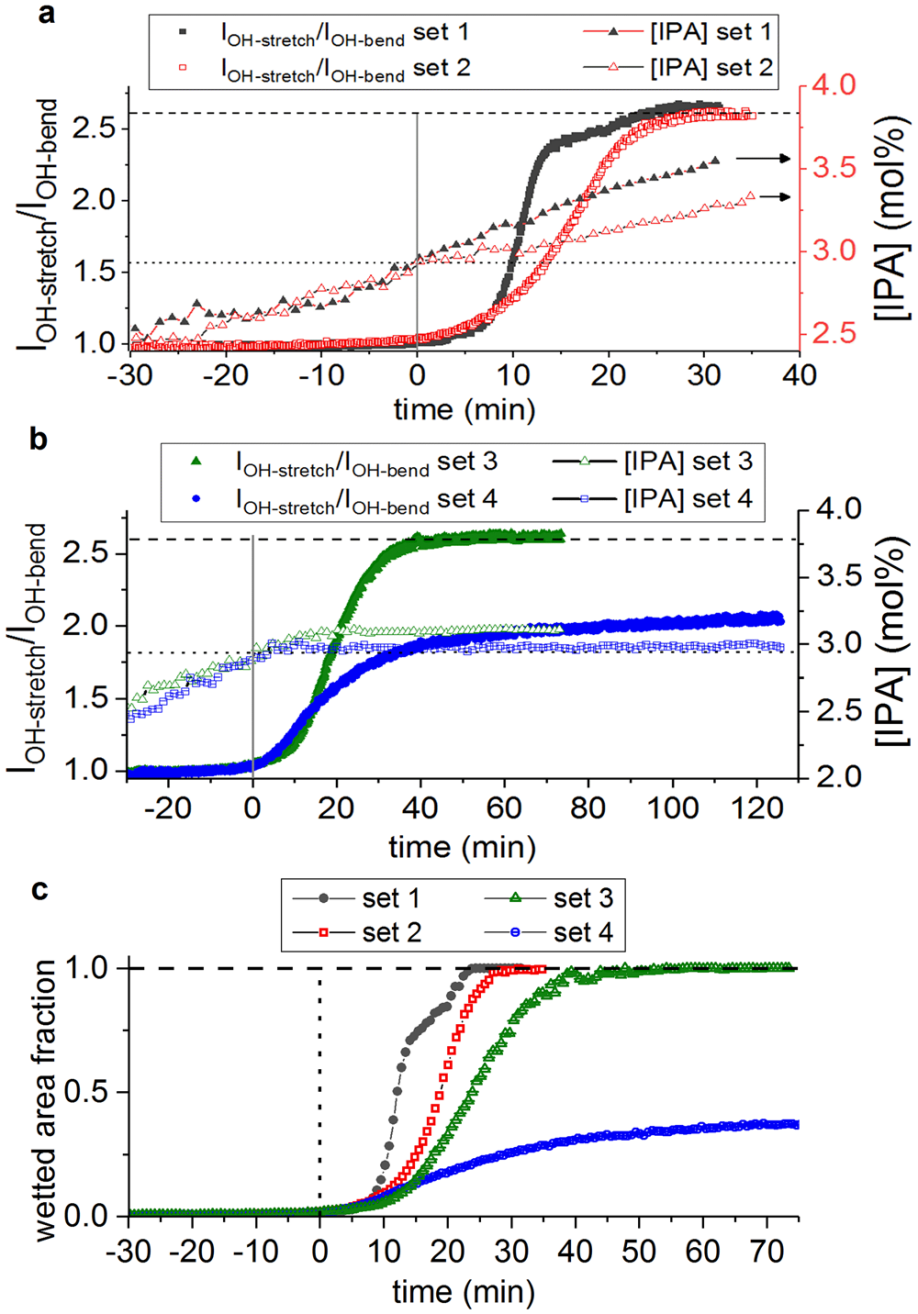
(**a,b**) Relative peak intensity ratio and corresponding IPA concentration profile for different experimental conditions. The dashed line at I_OH-stretch_/I_OH-bend_ = 2.6 (left axis) represents the peak intensity ratio corresponding to a fully wetted crystal. The dotted line marks the critical IPA concentration required to initiate the wetting (right axis). (**a**) Steady flow of IPA-saturated N_2_ is flown through the cell at two different flow rates (set 1: N_2_ flow rate = 350 ccm; set 2: N_2_ flow rate = 35 ccm), resulting in different concentration profiles and wetting speeds. (**b**) Stabilization of the IPA concentration at different levels around the critical concentration. (**c**) The comparison of wetting kinetics for all experiments, with wetted area fraction of the crystal calculated from the absorption intensities.

Without loss of generality, the dynamics of wetting transitions are studied using the 260 nm structures, as taller pillars give more pronounced differences between different wetting states (Fig. 3b). For IPA and water mixtures, there exists a very narrow window of compositions for which a wetting transition is observed, with ~2.8 mol% for the onset of superhydrophobic breakdown and 4 mol% for fully established Wenzel state. Therefore, in order to capture the wetting kinetics, a precise control of the IPA concentration is necessary, as elaborated in the Methods section. The dynamic change of the I_OH-stretching_/I_OH-bending_ intensity ratio as well as the instantaneous composition near the pillars are followed *in situ* by ATR-FTIR as shown in Fig. 6. FTIR cannot capture fast kinetics since the typical spectral acquisition time is in the range of 6–9 s. Therefore, different experiments are carried out to probe the relatively slow kinetics when concentration is close to the critical value. In the first set of experiments depicted in Fig. 6a, there is continuous condensation of IPA vapor for both conditions, but the condensation rate is slightly different. Similar features can be observed from both experiments. At low initial concentrations, the I_OH-stretching_/I_OH-bending_ intensity ratio remains close to 1.0 as corresponding to the Cassie-Baxter state. Then a sharp increase in the intensity ratio is found once the IPA concentration reaches a critical threshold of 2.93 mol%, the average value obtained from 9 experiments and marked by the black dotted line. The onset of wetting when the intensity ratio increases by 0.15 is taken as the time reference t = 0. Upon further increase of the IPA concentration, the relative peak intensity ratio gradually converges to 2.6, as marked by the dashed line in the graph, corresponding to the fully wetted Wenzel state. Note that for IPA-water mixtures, the peak intensity ratio value corresponding to full wetting is slightly higher than for pure water due to the minor contribution of IPA to the OH-stretching peak. Figure 6b depicts data sets for which the IPA concentration was stabilized at different values that are slightly higher (3.11 ± 0.1 mol%, set 3, Fig. 6b) or close to (2.98 ± 0.1 mol%, set 4, Fig. 6b) the critical concentration. Stabilization of the IPA concentration was achieved by switching off the gas flow to stop IPA condensation. Details can be found in the Methods section. Compared to the experiments shown in Fig. 6a, a much slower wetting kinetics has been observed, and the wetting transition was not complete even after hours when the IPA concentration got stabilized around the critical concentration (set 4).

When the I_OH-stretching_/I_OH-bending_ intensity ratio is in between the characteristic values for pure Wenzel or pure Cassie-Baxter states, it indicates a heterogeneous condition with mixed wetting states. Assuming a spatial heterogeneity of wetting with an area fraction x% of the surface in the Wenzel state, and 1 − x% in the Cassie-Baxter state. The area fraction can be calculated from the IR absorption intensities by a superposition of the contributions from different wetting states, as the total absorption intensity $I=x \% \,\ast \,{I}_{w}+(1-x \% )\,\ast \,{I}_{CB}$. The detailed calculations and experimental verifications can be found in Supporting Information. The time evolution of the wetted area fractions is plotted in Fig. 6c, and the wetting kinetics of all the experiments shown in Fig. 6a,b can be compared more quantitatively. Spontaneous wetting only starts when the IPA concentration is above the critical concentration, which was experimentally determined at 2.93 ± 0.07 mol%, corresponding to a surface tension of 38 mN/m and an intrinsic contact angle on flat surfaces *θ*_*c*_ = 91°. For IPA concentrations above the critical concentration (*θ* < *θ*_*c*_), surface energy is in favor of wetting more area, and wetting kinetics is expected to increase with the energy gain ~(1 − *cosθ/cosθ*_*c*_)^23^. For the very narrow range of liquid compositions depicted in Fig. 6, variations in contact angle are almost trivial, so wetting kinetics is only compared with IPA concentrations in this work. From Fig. 6, it is clear that higher IPA concentrations indeed result in faster wetting kinetics and less time to wet the entire surface. As pointed out in the work of Pirat *et al*.^25^, superhydrophobic breakdown near the transition regime occurs in a stepwise manner with a constant wetting front velocity (often referred to as ‘zipping’). Thus, the total wetted area ${{\rm{A}}}_{wet}\propto {({v}_{f}{\rm{t}})}^{2}$ with v_f_ the front velocity and t the elapsed time. When the wetted area fractions are plotted against t^2^ (figure S4 in Supporting Information), all experiments show an initial linear wetting regime. The average wetting front velocities and IPA concentrations can be extracted for this regime, as given in Table 3 (further details on the fitting can be found in Supporting Information). The obtained wetting speeds are on the order of tens of µm/s, and clearly slow down when the IPA concentration is approaching the critical concentration even though contact angle measurements do not reveal any differences. In literature, strongly diverging wetting front velocities ranging from 1 mm/s to 1.6 m/s have been reported for microstructures^23–25^. Such large dispersions of wetting velocities can be due to different energy gain for wetting of different structured surfaces. This also emphasizes the importance of wetting characterization techniques that can give more quantitative comparison than contact angle measurements.

**Table 3:** Wetting front velocities derived for the experimental sets shown in Fig. 6.

	V_f_ (µm/s)	${\bar{{\boldsymbol{[}}{\boldsymbol{I}}{\boldsymbol{P}}{\boldsymbol{A}}{\boldsymbol{]}}}}_{{\boldsymbol{l}}{\boldsymbol{i}}{\boldsymbol{n}}{\boldsymbol{e}}{\boldsymbol{a}}{\boldsymbol{r}}}$ (mol%)	${\bar{{\boldsymbol{\gamma }}}}_{{\boldsymbol{linear}}}$ (mN/m)
Set 1	45.9	3.19	37.1
Set 2	25.9	3.11	37.5
Set 3	17.9	3.08	37.6
Set 4	11.2	2.97	38.1

The front velocities are extracted from the experimental wetting curves by linear regression assuming A_wet_ = (v_f_ t)^2^. The IPA concentration and surface tension was taken as the average value over the linear part of the curve (Supporting Information).

As wetting proceeds, a slowing down of the kinetics is observed and the curves start to deviate from the t^2^ dependence (figure S4). Two possible phenomena can be responsible for this observation: (1) a change in the predominant wetting mechanism, e.g. wicking dynamics follows Washburn law instead of spreading at constant speeds^65^, or (2) slowing down due to interactions of the liquid fronts and merging of the wetted domains. Thus, a better understanding of the wetting mechanisms is necessary.

### Wetting mechanisms

**Figure 7:**
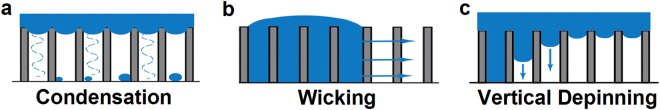
Schematics of (**a**) condensation in the pores, (**b**) wicking and (**c**) vertical depinning. The solid blue arrows indicate the movement of the liquid and the dashed lines illustrate evaporation and diffusion of IPA-vapor inside the pores.

The experimental results suggest a single critical composition, above which superhydrophobic breakdown occurs spontaneously. Yet, in theory three different wetting mechanisms are possible: (1) nucleation and growth of condensate on the structures, (2) wicking from a local infiltration area, and (3) vertical depinning. Figure 7 schematically illustrates these mechanisms. Owing to its high volatility, IPA might preferentially evaporate into the pores and condense on the structures. However, the spectra do not show any evidence of sudden increase of the IPA-concentration when wetting starts and measurements recorded on flat crystals show similar concentration profiles as on patterned crystals. These observations imply that formation of an IPA-rich condensate layer does not take place under the current experimental conditions. Therefore condensation events are not expected to have significant influence on the wetting in our experiments.

To distinguish between wicking and vertical depinning, the FDTS-layer is stripped at one end of the crystal using oxygen plasma to create a hydrophilic region of approx. 1 × 3 cm^2^. A mixture with IPA-concentration of 2.0 mol% is initially confined to this hydrophilic region with the contact line coinciding with the hydrophilic/hydrophobic interface. This configuration of a ‘liquid reservoir’ in contact with a superhydrophobic area allows to study the dynamics of wicking and excludes vertical depinning events. As the IPA-concentration is increased, the absolute intensity of the OH-bending band is monitored in time. This band is not affected by the presence of IPA, and an increase in peak intensity can be directly related to an increase in wetted area. If wicking takes place, the liquid is expected to invade the surface roughness according to a Washburn law (${{\rm{A}}}_{wet}\propto D{\rm{t}}$)^65^. For IPA concentrations up to 10 mol%, no significant increase of the wetted area is observed by ATR-FTIR, suggesting that wicking dynamics is negligible for the superhydrophobic surfaces used in our experiments. More experimental details can be found in Supporting Information. This also agrees with classical models, since a transition to hemi-wicking wetting takes place at contact angles that are much lower than the critical contact angles for Cassie-Baxter to Wenzel transitions^27,66^. As a consequence, the observed slowing down of the wetting kinetics can be attributed to the interaction between the wetting fronts and merging of wetted domains. Additionally, these findings imply that surface defects may induce local breakdown of the Cassie-Baxter state, but are not expected to give rise to significant spreading of the liquid front if the transition criterion is not satisfied. Vertical depinning is suggested to be the most plausible wetting mechanism for superhydrophobic breakdown. In our experiments, the obtained wetting front velocities are on the order of tens of µm/s, much slower than the reported values (mm/s~m/s) for micropillars^23–25^. Therefore, nanostructured surfaces can be highly potential for many applications because the higher density of pinning sites can slow down the superhydrophobic breakdown process.

### Wetting actuation and hysteresis

**Figure 8:**
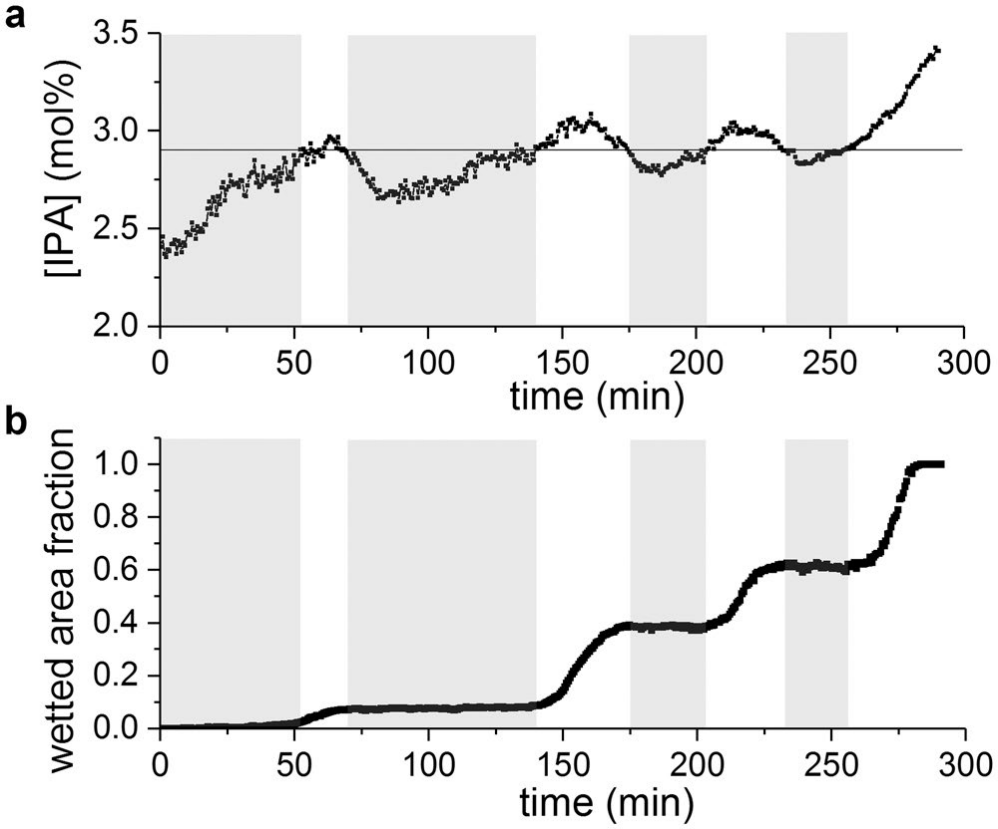
(**a**) IPA concentration profile oscillating around the critical concentration and (**b**) the corresponding wetted area fraction as a function of time. The horizontal black line marks the critical concentration for superhydrophobic breakdown. IPA concentrations above or equal to the critical concentration and the corresponding wetted area fraction are indicated with a white background for clarity.

Another important aspect for many applications of superhydrophobic surfaces is the feasibility to control and eventually reverse the wetting. By *in-situ* switching the gas flows in the liquid cell to modulate the condensation of IPA- or water vapor, the IPA concentration was artificially made oscillating around the critical IPA concentration. The real time IPA concentration and corresponding wetted area fractions are plotted as a function of time in Fig. 8. A wetting transition is initiated once the IPA concentration exceeds the critical IPA concentration (2.93 ± 0.07 mol%) and is stabilized with negligible hysteresis once the IPA concentration drops below this value. A monotonic increase of the wetted area fraction is observed, which implies that dewetting does not occur when the IPA concentration is lowered, irrespective of the remaining non-wetted area. This observation agrees with the works of Boreyko^67^ and Dorrer^43^, where the reverse Cassie-Wenzel transition is hindered due to excessive contact-line pinning. In our experiments, more significant pinning can be expected due to the small dimensions of the structures and large liquid-solid contact area in Wenzel state. Moreover, the hydrostatic pressure cannot be neglected for liquid films of few mm thick, and needs to be overcome to restore the superhydrophobic state. The experimental increase in liquid surface tension does not generate a sufficiently large driving force to overcome these energy barriers and transition to the Cassie-Baxter state. Large-area dewetting requires a much larger external energy input to create a vapor layer that lifts the drop (the Leidenfrost effect), as for example induced by laser-heating^68^ or high voltages^69^. The system can thus be stabilized in a mixed wetting condition and is very sensitive to small variations in liquid composition around the critical concentration. Complete wetting of the entire surface is achieved with an excellent control by increasing the IPA concentration above the critical concentration for prolonged times. This is the first demonstration of wetting actuation through external modulation of the atmosphere.

## Conclusions

ATR-FTIR is used for real time monitoring of wetting states and wetting transitions on immersed superhydrophobic nanopatterned surfaces. Differentiation between Wenzel, Cassie-Baxter and mixed wetting states is based on the relative intensity ratio of the water bands, which can be very accurately predicted by FDTD simulations. The transition criterion for superhydrophobic breakdown is determined as a function of structure geometry using both goniometric methods and ATR-FTIR. Classical models are found to overestimate the critical contact angles, and such deviations are postulated to be induced by the differences in surface state between the real sidewall surface of nanostructures and the flat surfaces used in contact angle measurements. Therefore, careful experimental assessment of the transition criterion is crucial in order to avoid a misinterpretation of the metastable superhydrophobic states and wetting dynamics. The dynamic process of superhydrophobic breakdown is found to proceed initially with constant speeds on the order of ~µm/s. The wetting dynamics is highly sensitive to small deviations near the critical concentration, which also underlines the importance of accurate transition criteria. For the first time, actuation of the Cassie-Baxter to Wenzel transition is demonstrated by *in-situ* modification of the liquid composition through atmosphere modulations. Wetting could be initiated and stopped with negligible hysteresis, although regeneration of the Cassie-Baxter state was impeded by contact line pinning and additional effects from hydrostatic pressure. Owing to its high sensitivity, ATR-FTIR can be a promising technique to characterize different superhydrophobic surfaces, including hierarchical structures. As the superhydrophobic breakdown is monitored on macroscopic (cm-sized) areas, it includes dynamic effects from multiple nucleation sites and interacting wetting fronts and is therefore very relevant to most real life applications.

## Data availability Statement

The datasets generated during and/or analyzed during the current study are available from the corresponding author on reasonable request.

## Electronic supplementary material

Supplementary information
