# Supplementary material for: In-situ ATR-FTIR for dynamic analysis of superhydrophobic breakdown on nanostructured silicon surfaces
Source: Sci Rep. 2018 Aug 2;8:11637. doi: 10.1038/s41598-018-30057-w (PMC6072713; doi:10.1038/s41598-018-30057-w)
Supplement: Supplementary file 1 — Supplementary information [file 41598_2018_30057_MOESM1_ESM.pdf]

## SUPPORTING INFORMATION

# In-situ ATR-FTIR for dynamic analysis of superhydrophobic breakdown on nanostructured silicon surfaces

Nandi Vrancken[1,2,*], Jiaqi Li[2,3], Stefanie Sergeant[2], Guy Vereecke[2], Geert Doumen[2], Frank Holsteyns[2], Chang Chen[2], Herman Terryn[1], Stefan De Gendt[2,4] and XiuMei Xu[2,*]

[1] Vrije Universiteit Brussel, Pleinlaan 2, 1050 Elsene, Belgium

[2] Imec, Kapeldreef 75, 3001 Leuven, Belgium

[3] Department of Physics and Astronomy, KU Leuven, Celestijnenlaan 200D, 3001 Leuven, Belgium

[4] KU Leuven, Celestijnenlaan 200, 3001 Leuven, Belgium

* corresponding authors: xiumei.xu@imec.be (X.M.)
nandi.vrancken@imec.be (N.V.)

## Effect of in-situ IPA concentration changes on the relative peak intensity ratio of the water bands

The relative peak intensity ratio of the water bands corresponding to a typical concentration profile for wetting kinetics tests is plotted in figure S1. The data shown is this figure is recorded on a FDTS-coated flat silicon crystal. Similar results are obtained on hydrophilic patterned samples (data not shown). Although the hydroxyl group present in IPA contributes to the OH-stretching band, this does not significantly affect the relative peak intensity ratio $I_{OH\text{-}stretch}/I_{OH\text{-}bend}$ in the concentration range covered by the experiments (0-4 mol%). A wider concentration range has already been assessed ex-situ in our previous work[1], where significant increase of this ratio has only been observed for IPA concentrations above 20 mol%. Thus, observed variations in the relative peak intensity ratio of the water bands can be directly correlated to changes in the wetting state.

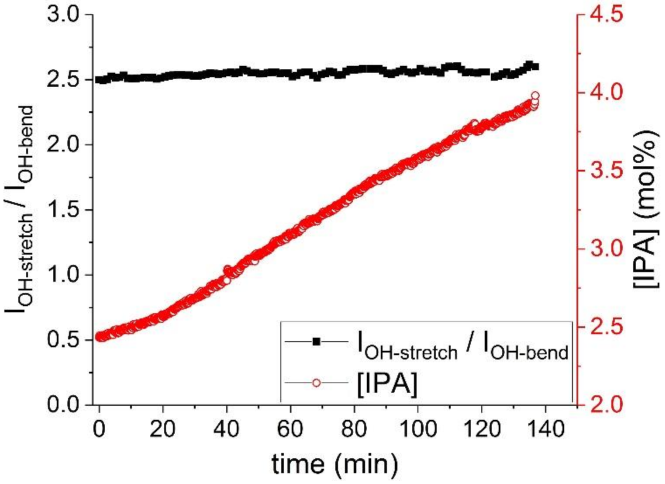


Figure S1. Variation of the relative peak intensity ratio of the water stretching and bending bands upon changing the IPA concentration on a FDTS-coated flat silicon sample.

## Determination of the transition criterion with ATR-FTIR

A first estimate of the transition criterion for superhydrophobic breakdown is obtained from contact angle measurements. This value corresponds to a concentration that leads to full wetting in a very short timescale. ATR-FTIR can measure over longer timescales and can therefore yield a more accurate value for the onset of the wetting transition. IPA/water mixtures with a concentration well below the critical concentration found with contact angle measurements are injected in the liquid cell. Then, the IPA concentration is slowly increased by flowing 35 ccm of $N_2$ gas saturated with IPA through the cell. The real-time concentration near the surface and relative peak intensity ratio of the water bands are monitored in situ. The obtained wetting curve is depicted in figure S2 for the 78 nm pillars. The onset of wetting is defined as the point where the relative peak intensity ratio of the water bands increased by 0.15 and is shown in the graph as the crossover between the vertical dashed line and the top horizontal black line. This threshold is an arbitrary chosen value to eliminate wetting effects from die lines and other surface defects. The bottom horizontal line marks the corresponding critical IPA concentration. Similar curves are recorded for pillars with different dimensions, with the absolute values corresponding to the initial $I_{OH\text{-stretching}}$ / $I_{OH\text{-bending}}$ ratio and the critical IPA concentration depending on the pillar geometry. The critical contact angle is determined through contact angle measurements on a flat FDTS-coated silicon surface using a mixture with the same IPA concentration as determined with ATR-FTIR.

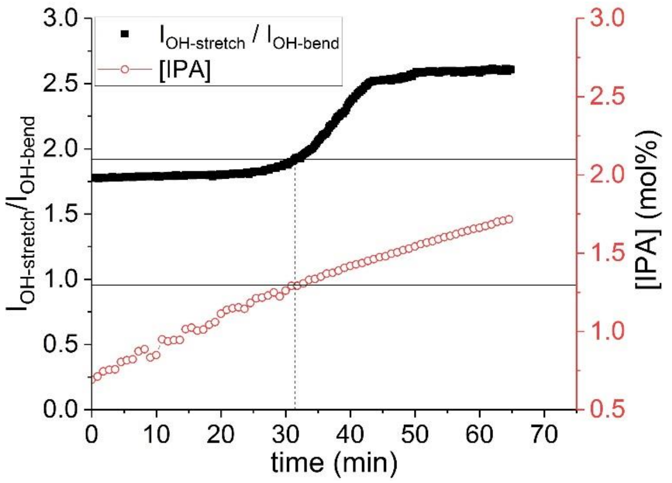


Figure S2: determination of the transition criterion for the 78 nm pillars. The top solid horizontal line represents an increase of the relative intensity ratio by 0.15 with respect to the initial value. The bottom solid horizontal line marks the corresponding IPA-concentration. This is the critical concentration for superhydrophobic breakdown.

## Assessment of the wetted area fraction

Assessment of the total wetted area fraction is feasible based on the obtained relative peak intensity ratio of the water bands and the wetting spectra corresponding to pure Wenzel and Cassie-Baxter states on the same surface. This approach yields an average wetting state of the surface, although no information is obtained locally.

Consider the case of a partially wetted crystal. The absolute peak intensities of the OH-stretching and OH-bending bands can be calculated as the superposition of the contributions from the wetted and the non-wetted areas:

$$I_{OH-bend} = x * I_{OH-bend,W} + (1-x) * I_{OH-bend,CB} \quad (1)$$

$$I_{OH-stretch} = x * I_{OH-stretch,W} + (1-x) * I_{OH-stretch,CB} \quad (2)$$

Here, x is the area fraction in Wenzel state and (1-x) is the area fraction in Cassie-Baxter state. The subscripts *W* and *CB* are used to denote the absolute peak intensities of reference spectra corresponding to the pure Wenzel and Cassie-Baxter states. Thus, the relative intensity ratio of the water peaks can be written as:

$$r = \frac{I_{OH-stretch}}{I_{OH-bend}} = \frac{x\, I_{OH-stretch,W} + (1-x) * I_{OH-stretch,CB}}{x * I_{OH-bend,W} + (1-x) * I_{OH-bend,CB}} \quad (3)$$

And thus:

$$x = \frac{I_{OH-stretch,CB} - I_{OH-bend,CB} * r}{r * I_{OH-bend,W} - r * I_{OH-bend,CB} - I_{OH-stretch,W} + I_{OH-stretch,CB}} \quad (4)$$

Experimental validation of this equation is done by controlled partial stripping of the FDTS layer of the superhydrophobic crystals. Heterogeneous samples are prepared by partly covering the sample with a protective layer and subjecting the sample to 6 seconds of $O_2$-plasma at 100 W. This treatment removes the FDTS layer on the uncovered area (thus rendering this area hydrophilic), whereas the protected area remains superhydrophobic. The crystals are then mounted in the liquid cell and covered with water. The wetted fraction equals the hydrophilic fraction of the crystal and can thus be directly related to the measured relative peak intensity ratio of the recorded water spectrum. Two samples have been prepared with 25% and 65% hydrophilic area fraction, respectively. The resulting heterogeneous wetting states were found to be stable in time (measurement times up to 1h). No significant variations were observed when turning the crystal by 180°, indicating that the relative

position of the wetted zone does not notably affect the recorded spectrum. The experimental data is plotted in figure S3. A good agreement is found with the theoretical curve based on equation 4.

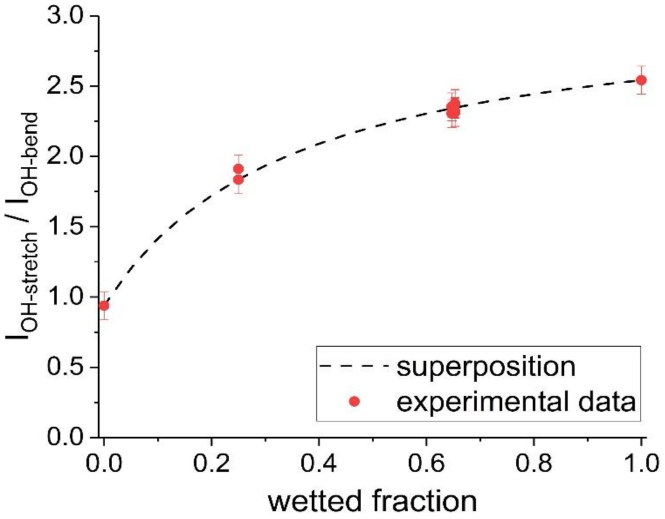


Figure S3. Relative peak intensity ratio as a function of the actual wetted area fraction. The experimental data is obtained by controlled partial removal of the FDTS-coating on the patterned samples. The black dashed line corresponds to the theoretical peak intensity ratio based on the superposition principle (equation 4).

## Wetting kinetics: extraction of the front velocity

Wetting on microstructures has been shown to follow a stepwise mechanism with a constant front velocity for solid/liquid couples near the critical transition criterion.[2] In case of a single infiltration point on the ATR-crystal, the total wetted area is calculated as $A_{wet} = (v_f\ t)^2$ with $v_f$ the front velocity and t the elapsed time. Figure S4 depicts the wetted area fraction ($A_{wet}/A_{total}$) as a function of $t^2$ for the four experimental sets discussed in the main text. Three different wetting regimes can be observed: (1) initiation stage, (2) linear regime, and (3) slowing down of the wetting kinetics due to merging of the liquid fronts. The data used for linear fitting are indicated in red. The wetting front velocity can be calculated from the slope of this linear part as $v_f = \sqrt{(A_{wet}/t^2)}$ where $A_{wet}/t^2$ equals the slope multiplied by the total surface area. Wetting front velocities have been extracted from 9 different experiments in total.

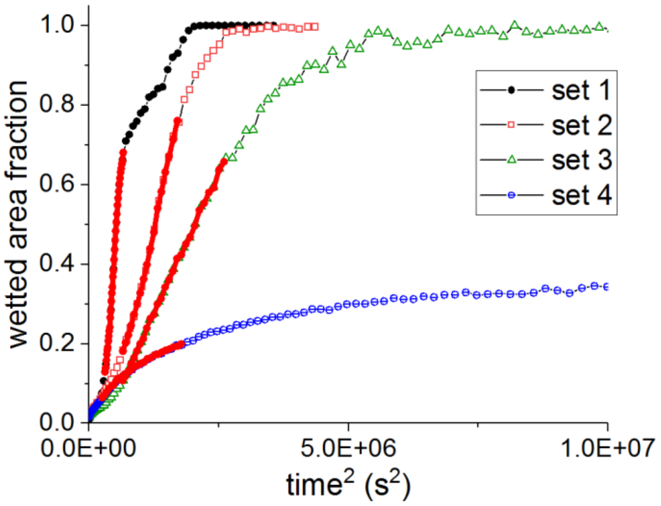


Figure S4. Extraction of the front velocity from the four data sets discussed in the main text. The linear regions used for fitting are indicated in red.

## Wetting mechanisms: wicking test

In order to separate the contributions from wicking and vertical depinning, a superhydrophobic ATR-crystal was partially stripped to create a hydrophilic reservoir. Part of the crystal is covered with a protective layer and subsequently the crystal is subjected to $O_2$-plasma treatment at 100 W for 6 seconds. The uncovered side is rendered hydrophilic by $O_2$-stripping, whereas the covered side remains superhydrophobic. A visual illustration of this behavior is given in figure S5 for FDTS-coated flat silicon and a patterned sample.

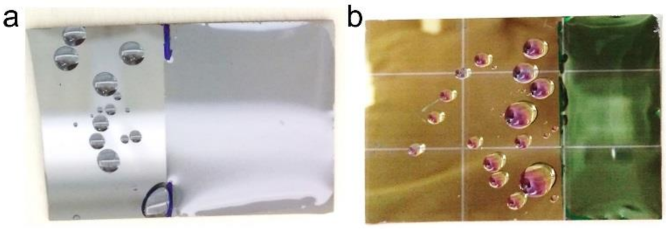


Figure S5. Partially stripped samples. (a) flat silicon dummy, (b) patterned sample. The left hand side of the crystals is superhydrophobic, whereas the right hand side has been exposed to $O_2$-plasma treatment to remove the FDTS-layer.

After mounting the crystal in the liquid cell, an IPA/water mixture with a concentration of 2.0 mol% is injected on the hydrophilic part of the crystal. The liquid forms a drop with the contact line coinciding with the hydrophilic/hydrophobic interface. No spreading onto the hydrophobic side is observed. As liquid is present in a reservoir in contact with the FDTS-coated pillars and does not rest on top, Wicking is the only possible wetting mechanism in this experiment. The IPA content of the liquid is increased by flowing IPA-saturated nitrogen through the cell at a rate of 35 ccm. The real time composition and the absolute peak intensity of the OH-bending band as a function of time are plotted in figure S6. The relative peak intensity ratio of the water peaks is not an accurate measure in this case, as a ratio of ~2.6 $\pm$ 0.1 is obtained for Wenzel wetting, irrespective of the total wetted area. However, the absolute intensity of the OH-bending band is also not affected by the presence of IPA, and an increase in peak intensity can be directly related to an increase in wetted area. The dashed line at 0.29 indicates a typical intensity value corresponding to a fully wetted crystal (this value may vary slightly depending on the crystal quality and the experimental conditions). The initially observed value of 0.07 corresponds to the configuration in which the hydrophilic part is wetted and no liquid is present on the hydrophobic part. A small increase is observed after 23.5 min, corresponding to an IPA concentration of 3.1 $\pm$ 0.2 mol%. This value is close to the previously reported critical concentration for superhydrophobic breakdown in the bulk liquid. The increase is attributed to flattening of the drop edges and possible vertical depinning of the liquid in between the underlying pillars. No further significant increase is observed for IPA concentrations up to 10 mol%, suggesting that the contribution from Wicking is negligible.

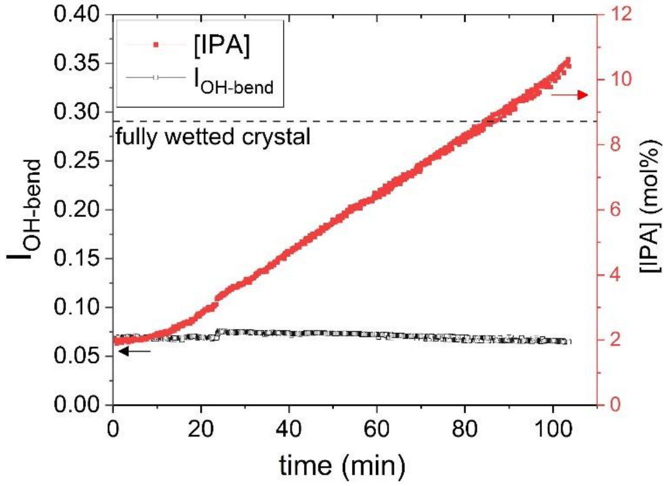


Figure S6. Intensity of the OH-bending band and IPA-concentration as a function of time, measured on a partially FDTS-coated crystal with liquid injected on the hydrophilic side.

## References


1. Vrancken, N. *et al.* Superhydrophobic Breakdown of Nanostructured Surfaces Characterized in Situ Using ATR − FTIR. *Langmuir* **33,** 3601–3609 (2017).
2. Pirat, C. *et al.* Multiple time scale dynamics in the breakdown of superhydrophobicity. *EPL* **81,** 1–6 (2008).
